# Reflecting microscope system with a 0.99 numerical aperture designed for three-dimensional fluorescence imaging of individual molecules at cryogenic temperatures

**DOI:** 10.1038/srep12833

**Published:** 2015-08-04

**Authors:** H. Inagawa, Y. Toratani, K. Motohashi, I. Nakamura, M. Matsushita, S. Fujiyoshi

**Affiliations:** 1 Department of Physics, Tokyo Institute of Technology, Meguro, Tokyo, 152-8550, Japan; 2 Japan Science and Technology Agency, PREST, Kawaguchi, Saitama, 332-0012, Japan

## Abstract

We have developed a cryogenic fluorescence microscope system, the core of which is a reflecting objective that consists of spherical and aspherical mirrors. The use of an aspherical mirror allows the reflecting objective to have a numerical aperture (NA) of up to 0.99, which is close to the maximum possible NA of 1.03 in superfluid helium. The performance of the system at a temperature of 1.7 K was tested by recording a three-dimensional fluorescence image of individual quantum dots using excitation wavelengths (*λ*_ex_) of 532 nm and 635 nm. At 1.7 K, the microscope worked with achromatic and nearly diffraction-limited performance. The 1/*e*^2^ radius (*Γ*) of the point spread function of the reflecting objective in the lateral (*xy*) direction was 0.212 ± 0.008 μm at *λ*_ex_ = 532 nm and was less than 1.2 times the simulated value for a perfectly polished objective. The radius *Γ* in the axial (*z*) direction was 0.91 ± 0.04 μm at *λ*_ex_ = 532 nm and was less than 1.4 times the simulated value of *Γ*. The chromatic aberrations between the two wavelengths were one order of magnitude smaller than *Γ* in each direction.

The most detailed understanding of living cells is derived from molecular studies of the local activities that occur in a cell. The experimental requirements for molecular-level observations are a spatial resolution that is high enough to resolve molecular arrangements and molecular specificity that enables identification of the chemical species involved. Among the experimental methods being used to study nanometre-scale cellular structures, cryogenic electron tomography is the most advanced in terms of spatial resolution (4-5 nm). However, the 4-5 nm resolution is insufficient for the direct assignment of macromolecules^1^. By contrast, visible fluorescence microscopy enables the assignments of molecules in a living cell with molecular precision using site-selective fluorophore labelling via either genetically-encoded protein tags^2^ or unnatural amino acids^3^,^4^. Furthermore, techniques have been developed in the field of single-molecule fluorescence imaging to overcome diffraction limit of visible light. The position of individual fluorophores that are spatially well-isolated on an image can be determined as the position of the centroids of each fluorescence spots with 1 nm precision. The technique is called fluorescence imaging with one nanometre accuracy (FIONA)^5^,^6^. To determine the overall 3D arrangements of many fluorophores whose fluorescence spots overlap each other, a technique called superresolution fluorescence microscopy is employed^7^,^8^,^9^. Fluorophores bound to a target molecule are stochastically switched on at different times, and the arrangements of multiple photo-switched fluorophores are determined by processing multiple frames of the fluorescence image independently. Under ambient conditions, target molecules will move before the necessary number of frames is acquired; thus, the localisation precision of superresolution fluorescence microscopy has been limited to ca. 50 nm^10^,^11^,^12^,^13^, which clearly differs from the high precision of FIONA (1 nm). Therefore, superresolution fluorescence imaging with a truly molecular-level precision requires sample immobilisation.

To this end, the method that we have pursued is cryogenic (cryo-) immobilisation. Cryo-immobilisation that employs a rapid freezing below a glass transition temperature of 135 K is called vitrification and preserves a near native state of structures in vitrified water^14^,^15^,^16^. Cryo-fluorescence microscopy of a vitrified sample can be performed for several hours and they may provide a detailed snapshot of the moment when a cell was frozen. Several attempts have been made to apply fluorescence microscopy to vitrified biological samples^17^,^18^,^19^,^20^; however, these pioneering works have been suffered from technical difficulties. For most cryo-fluorescence microscope studies, the sample was cooled below the devitrification temperature while the objective lens remained at ambient temperatures. In such a configuration of a microscope, it is difficult to simultaneously obtain high mechanical stability and a high numerical aperture (NA). In our microscope, both the sample and the objective lens are mounted on a rigid holder and immersed in superfluid helium at a few K. Superfluid helium is also used as a cryogen in other high spatial resolution microscopy, including a cryo-electron microscope^21^. With this construction, we have passively reduced the mechanical drift between the sample and the objective lens to <1 nm/10 min^22^. Here, we demonstrate a mechanically stable cryo-fluorescence microscope equipped with a reflecting objective that has an ultimate design of NA of 0.99. The major problem with our previous microscope was the lack of a high-NA objective that works in superfluid helium. The problem has been solved in the present work. In the future, the microscope developed in the present work will enable us to conduct cryo-superresolution fluorescence imaging with a molecular-level localisation precision similar to that of FIONA.

In the last decade, we have developed several reflecting objectives for single-molecule cryo-imaging^23^,^24^,^25^,^26^,^27^,^28^,^29^. The first one (see Fig. 1b) has an NA of 0.58. Using this reflecting objective in a cryo-fluorescence microscope, the localisation precision was 3 nm in the lateral (*xy*) direction and 18 nm in the axial (*z*) direction^29^. The *xy* precision had reached the macromolecular level. The *z* precision has to be improved to perform nanoscale 3D imaging. The localisation precision in a certain direction is proportional to the width of the point spread function (PSF) in that direction. The width of the PSF in the *z* direction is proportional to *nλ*/NA^2^ = *λ*/(*n*sin^2^*θ*); thus increasing the NA of the reflecting objective can decrease the width of the PSF. In the above relation, *n* represents the refractive index of the immersion medium (superfluid helium in the present work), *λ* is the wavelength of the light, and *θ* is the aperture angle of the objective lens. Due to spherical aberration, NA of reflecting objectives has remained approximately 0.6 at maximum in ambient consitions^30^,^31^. In the current work, we have developed a reflecting objective with an NA of 0.99 in superfluid helium using an aspherical mirror that can be implemented in an aberration-free design. The NA of 0.99 is almost identical to the maximal NA of a dry objective lens in superfluid helium (NA = *n*sin*θ* ≤ 1.03, *n* = 1.027 for superfluid helium^32^). Using the high-NA reflecting objective, we have constructed a reflecting microscope system. At cryogenic temperatures, this fluorescence microscope exhibited achromatic and nearly diffraction-limited performance. The performance of the cryo-microscope system is estimated to be sufficient for observing 3D molecular arrangements.

## Results

### Design and simulation of the aspherical reflecting objective

**Figure 1:**
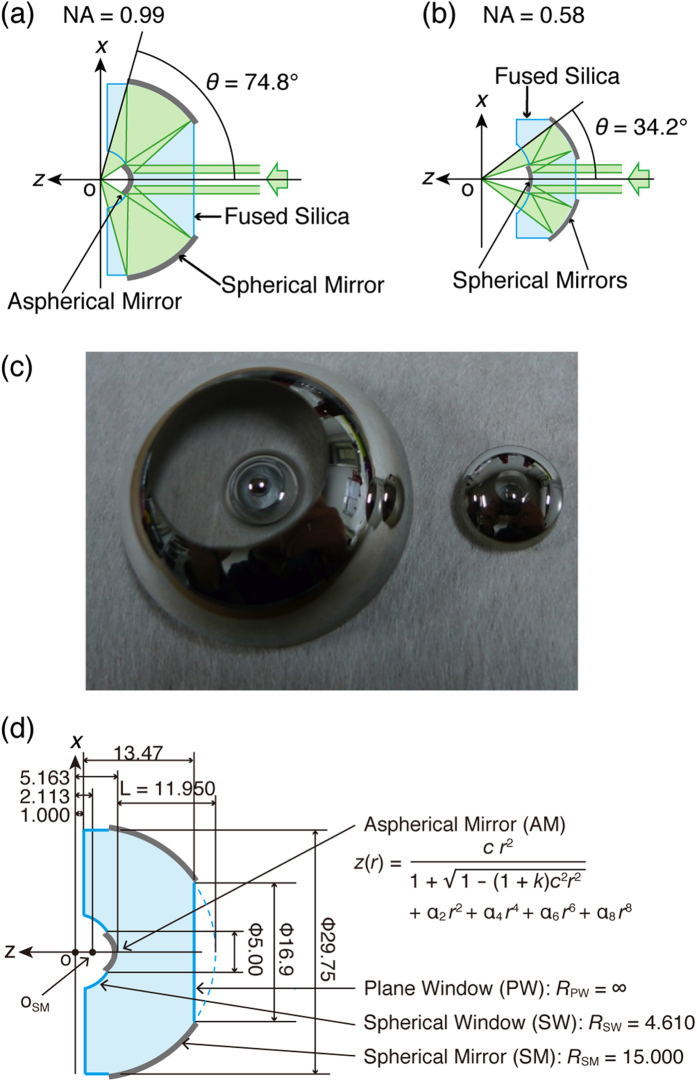
(**a**,**b**) Optical design of the aspherical (**a**) and spherical (**b**) reflecting objectives. (**c**) Photograph of the aspherical (left) and spherical (right) reflecting objectives. (**d**) Dimensions of the aspherical reflecting objective.

Figure 1a shows the optical design of the aspherical reflecting objective with an NA of 0.99 in superfluid helium. Fused silica was chosen as the material for the reflecting objective because of its high transparency from ultraviolet to near-infrared wavelengths as well as its small thermal expansion coefficient^23^,^25^. The spherical and aspherical mirrors were produced by vacuum deposition of aluminium onto polished fused-silica surfaces. We optimised the dimensions of the aspherical reflecting objective using commercial software (Zemax-EE) (see the Methods section for details regarding the optimisation procedure.) The specifications and the optimised dimensions are provided in Table 1 and Fig. 1c, respectively. As a result of the optimisation, the aperture angle *θ* of the aspherical reflecting objective, 74.8° became almost double the angle of the spherical reflecting objective of Fig. 1b (34.2°). The NA (=*n*sin*θ*) of 0.99 is very close to the maximum value of 1.027 (=*n*) in superfluid helium^32^.

**Figure 2:**
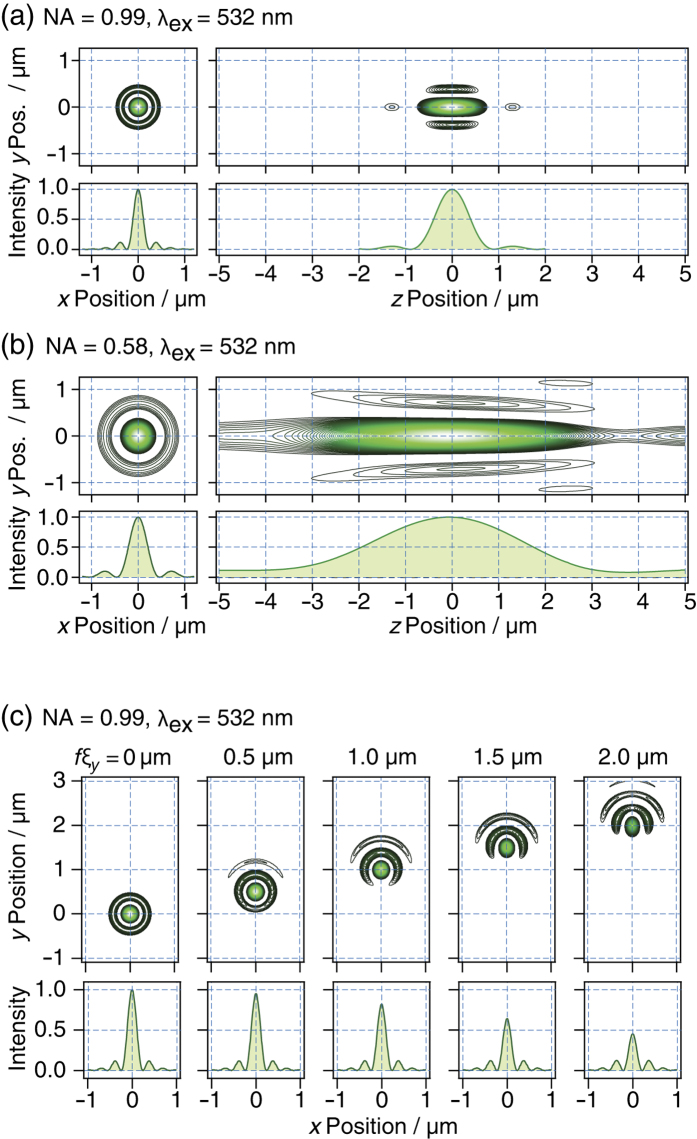
(**a**,**b**) Zemax simulation of the three-dimensional point spread function (PSF) of the aspherical (**a**) and spherical (**b**) reflecting objectives at *λ*_ex_ = 532 nm. The intensity plots along the horizontal lines at *y* = 0 μm are shown below each PSF. (**c**) Zemax simulation of PSFs used to evaluate the field of the view of the aspherical reflecting objective at *λ*_ex_ = 532 nm. The intensity plots along the horizontal lines at *y* = *fξ*_y_ are shown below each PSF.

We have simulated the 3D PSF of the aspherical (Fig. 2a) and spherical (Fig. 2b) reflecting objectives in superfluid helium. All of the simulations in the present work were conducted using the Huygens method in Zemax. The lateral 1/e^2^ radius of the central disc (*Γ*_xy_) of the aspherical objective with NA = 0.99 was 0.182 μm at *λ* = 532 nm, which is two times better than the *Γ*_xy_ of the spherical objective with NA = 0.58. The *Γ*_z_ of the aspherical objective was 0.64 μm at *λ* = 532 nm, which is a fivefold improvement compared with the *Γ*_z_ of the spherical objective. The field of view (FOV) of the objective lens was evaluated using an optical simulation. Figure 2c shows the simulation of the 2D PSF of the aspherical reflecting objective on the focal plane as a function of the incident angle of the parallel beam with respect to the principal ray, *ξ*. Clearly, the central disc in the PSF is distorted at *fξ* > 1.5 μm, where *f* is the focal length of the aspherical reflecting objective. The FOV of the aspherical reflecting objective is estimated to be 2 × 2 μm^2^. To obtain a microscope with a sufficient FOV for imaging cellular structures, we have introduced a 3D cryo-sample scanning system into the microscope (see the next section).

### Setup of the cryo-fluorescence reflecting microscope

**Figure 3:**
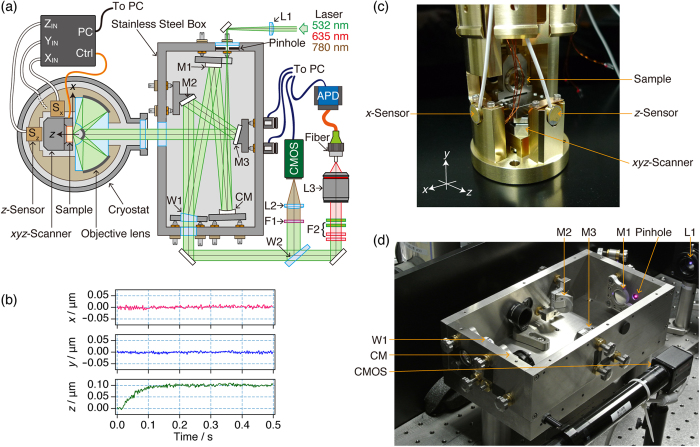
(**a**) Setup of the cryogenic fluorescence microscope using an aspherical reflecting objective. (**b**) The temporal behaviour of the *xyz*-position of the cryo-stage at 1.7 K after receiving a command to increment the *z* direction by 0.1 μm. (**c**,**d**) Photographs of the sample holder and the stainless steel box, respectively. The lid of the box was removed when photograph (**d**) was taken.

Figure 3a shows the reflecting microscope that was designed for the 3D imaging of individual molecules at a few K. The details of the setup are described in the Methods section. The light sources were continuous-wave diode lasers of *λ* = 532, 635, and 780 nm. Laser beams of 532 and 635 nm were used to excite the sample. To avoid sample damage from photo-excitation, the 780 nm wavelength, which is non-resonant with the absorption of the sample, was employed to monitor the incident angle of the excitation laser beams. The three laser beams were focused onto a light-source pinhole aperture with a lens (L1). The diffracted laser light was collinearly collimated using a concave mirror (CM). The collimated beams were reflected using the front surface of a 1°-wedge window of CaF_2_ (W1) and continued to travel into a cryostat of superfluid helium.

To minimise the mechanical fluctuations caused by thermal drift and the finite rigidity, the core elements of the confocal setup, i.e., the six optical elements starting from the pinhole to mirror M3 in Fig. 3a, were placed in a stainless-steel box. All of the entrances for light were sealed with CaF_2_ windows to make the box airtight. The laser beams left the box as parallel light to propagate to the cryostat. The mechanical drift between the box and the cryostat does not degrade the fluorescence images. The laser beams were focused on the sample surface using the aspherical reflecting objective in superfluid helium. The high mechanical stability was obtained by fixing the reflecting objective and the sample to a rigid holder and inserted into superfluid helium. The *xyz* position of the sample was controlled by a piezo-driven 3D stage using a closed-loop feedback system in which an error signal was generated by capacitive position sensors for the *xyz* directions (*S*_x_, *S*_y_, and *S*_z_). The performance of the closed loop system was examined at 1.7 K (Fig. 3b). At *t* = 0 s, we moved the *z* position by 0.1 μm. The movement was completed in at least 0.2 s, and the standard deviations of the *xyz* positions in the time region of 0.2 s to 0.5 s were 4.6 nm (*x*), 3.7 nm (*y*), and 4.3 nm (*z*) at the sampling rate of 500 Hz. The FOV of the cryo-microscope was 150 × 15 × 150 μm^3^ in the *x* × *y* × *z* directions, respectively (see Methods section). The fluorescence from the sample excited at the wavelengths (*λ*_ex_) of 532 and 635 nm was collected with the reflecting objective, passed through the two wedge windows W1 and W2, separated completely from the excitation light by a stack of long-pass and notch filters (F2), coupled with a lens (L3) to a multimode fibre, and counted using an avalanche photo-diode detector (APD). 3D cryo-fluorescence images were obtained by plotting fluorescence photon counts as a function of the *xyz* position of the sample. Photographs of the sample holder and the stainless steel box are shown in Fig. 3c,d.

### 3D cryo-fluorescence imaging of individual quantum dots at a few K

**Figure 4:**
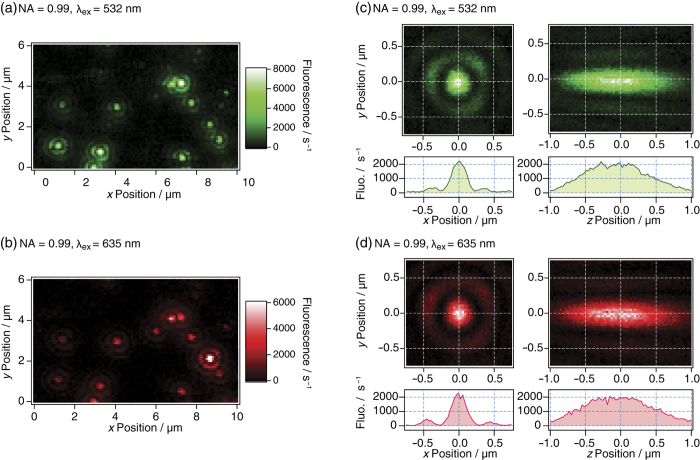
(**a**,**b**) Two-dimensional fluorescence image of individual quantum dots at 1.7 K. The signals from the area *x* × *y* = 10 × 6 μm^2^ (100 × 60 pixel^2^) were obtained using excitation wavelengths *λ*_ex_ = 532 (**a**) and 635 (**b**) nm. (**c**,**d**) Three-dimensional fluorescence images of a single dot at 1.7 K. The left side shows an *xy* section of 1.5 × 1.5 μm^2^ (50 × 50 pixel^2^), and the right side shows a *zy* section of 2.0 × 1.5 μm^2^ (67 × 50 pixel^2^). The fluorescence intensities along the horizontal lines at *y* = 0 μm are shown below each image. The exposure time was 0.1 s/pixel and the stage scanning time without the exposure time was 0.16 s/pixel. The excitation radiation was circularly polarized with an intensity of 80 W cm^–2^ (**a**), 120 W cm^–2^ (**b**), 70 W cm^–2^ (**c**), and 85 W cm^–2^ (**d**).

The performance of the reflecting microscope was examined at 1.7 K by taking fluorescence images of individual quantum dots. Quantum dots are widely used as fluorescence probes for cellular imaging^33^,^34^. The quantum dot (Qdot705) can be excited at *λ*_ex_ = 532 and 635 nm, and it emits fluorescence at approximately 705 nm. Figure 4 shows a 2D fluorescence image of the dots at 1.7 K in an *x* × *y* region of 15 × 6 μm^2^ obtained by the excitation at 532 (a) and 635 nm (b). Several tens of individual dots were observed as spatially isolated spots in these images. Every dot appeared on both fluorescence images at the same position, which proves the chromatic aberration-free design of the microscope. A 3D fluorescence image of a single dot at 1.7 K was observed using the excitation wavelengths of 532 (Fig. 4c) and 635 nm (Fig. 4d). In these figures, the images on the left are of an *xy* section of 1.5 × 1.5 μm^2^ and those on the right are of a *zy* section of 1.5 × 2 μm^2^.

In the microscope setup shown in Fig. 3a, the core of the multimode fibre was used as a detection pinhole aperture. The mode field diameter of the fibre was 50 μm, which is six times larger than that of the expected fluorescence spots on the fibre (approximately 8 μm). Thus, the fluorescence images reflect the 3D PSF of the excitation laser light around the focal point. The 1/e^2^ radius of the central disc (*Γ*) was evaluated by fitting the imaging to a Gaussian function. The average values of the radii *Γ* evaluated for five individual dots and the simulations are summarised in Table 2. The radii *Γ* of the reflecting objective with NA = 0.99 are two times (*Γ*_xy_) and four times (*Γ*_z_) smaller than those of the previous reflecting objective with NA = 0.58. A comparison with a simulation of a perfectly polished reflecting objective showed that the observed *Γ*_xy_ was 1.16 times that of the simulated *Γ*_xy_, and the observed *Γ*_z_ was 1.4 times that of the simulated *Γ*_z_. Moreover, the colour registrations of the 3D images between the two wavelengths (Fig. 4c,d) were *Δx*_RG_ = 7 ± 1 nm, *Δy*_RG_ = 13 ± 1 nm, and *Δz*_RG_ = 16 ± 4 nm, and they were an order of magnitude smaller than *Γ*. In summary, these results confirm that the reflecting microscope worked at cryogenic temperatures with achromatic and nearly diffraction-limited performance.

We have developed a new reflecting objective with a high NA of 0.99 and using this objective a microscope setup was built for 3D fluorescence imaging of individual molecules in superfluid helium. To obtain a good quality image using the high-NA reflecting objective, the wavefront error of the laser light must be suppressed to less than *λ*/4. The surface irregularity of the reflecting objective fulfils this requirement (see the Methods section). Because the reflecting objective was used in superfluid helium, cooling down of the objective to 1.7 K would introduce additional wavefront errors of the light. Nevertheless the microscope setup as a whole exhibited nearly diffraction-limited performance for samples in superfluid helium (Fig. 4). In addition, the mechanical stability of the cryo-microscope was comparable to the size of macromolecules (Fig. 3b). The FOV of the cryo-microscope was 150 × 15 × 150 μm^3^ in the *x* × *y* × *z* directions, respectively. The smaller range in the *y* direction is merely due to the scan range limit of the cryo-scanner used in the *y* direction and can be easily extended to 150 μm (see the Methods section).

## Discussion

The essential geometrical information of multi-protein complex is a relative distance vector between two proteins constituting the complex. If the two proteins are selectively labelled with two fluorophores that absorb light at different wavelengths, this information can be obtained using our cryo-microscope as the distance between the two fluorophores. Let us start with the experimental results of position measurements of single Qdot705 using two different excitation wavelengths (532 and 635 nm) for our old cryo-microscope in which the objective was the spherical reflecting objective with a low NA of 0.58 (Fig. 1b). With the total number of the collected photons (*N*) of 10^6^ in the total acquisition time of one hour, the precision of the distance between red and green fluorophores was estimated to be 3 nm in the lateral (*xy*) and 18 nm in the axial (*z*) directions^29^. The next step is to compare the low-NA microscope with NA = 0.58 (Fig. 1b) with the present one with NA = 0.99 (Fig. 1a). Under the same condition of the total acquisition time and the energy flux density of the laser excitation, the precision is proportional to 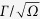, where *Ω* is the solid angle of an objective^35^. As a result of the reduction of *Γ*, the localisation precisions obtained with the aspherical reflecting objective (Fig. 1a) will improve by a factor of two in the *xy* directions and by a factor of four in the *z* direction. In addition, the increase of *N* quadruple by improvement of *Ω* will improve the precision in all the three direction by a factor of two. Consequently, the 3D precisions of the distance between two fluorophores measured by the microscope setup of the present work (Fig. 3a) are estimated to be 1 nm in the *xy* direction and 2 nm in the *z* direction.

One of the goals in the future of developing cryo-fluorescence microscope is to observe molecular arrangement in cells with molecular-level precision. In the case of pentacene in a *p*-terpheyl crystal, the linewidth of the 0-0 transition of an ensemble of pentacene is broadened by local variation of the crystal filed. At cryogenic temperature, the linewidth of individual pentacene molecules is several orders of magnitude narrower than the inhomogeneous width of the ensemble. The spectral selection of a single pentacene molecule is possible by tuning the wavelength of the excitation laser in resonance with one molecule at one time^36^. By using other photo-processes, two groups demonstrated 2D cryo-superresolution imaging of cells in 2014. R. Kaufmann *et al.* used reversible photo-bleaching of standard green fluorescent proteins (GFP)^18^ that occurs uniquely below 100 K^37^,^38^. Y.W. Chang *et al.* used the photo-switching of photoactive GFP^20^, a process similar to that occurs at ambient temperatures^7^,^8^. As a result, a single fluorophore in cells at cryogenic temperatures can be selected from a large number of the fluorophores in the laser focal volume. However, localisation precisions of the above-mentioned works were limited to one hundred nanometres. In the present work, we made a step forwards but still a number of technical hurdles have to be overcome. For example, it takes too long time to measure an arrangement of multiple fluorophores with a high precision. With the present confocal microscope, the nanometre-scale determination of the relative distance between two fluorophores needs to collect 10^7^ photons, which corresponds to one hour of the acquisition time. It would take days to obtain an arrangement of multiple fluorophores. This is because an image is taken by a confocal scanning of a 1D detector of an APD. In single-molecule detections at cryogenic temperatures, the fluctuation of the fluorescence photon counts with time is a noise due to photo-blinking^39^, typically ten times larger than the shot noise of collected photons. If the 1D detector of APD is replaced by a 2D imaging detector leaving all the other optical elements intact (Fig. 3a), photo-blinking will not contribute to the noise of the photon distribution over the image^36^,^40^,^41^,^42^. Then, the error in the lateral (*xy*) localisation is close to the shot noise limit, 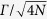
35,43. Under the condition listed in Table 2 (*Γ*_xy_ = 0.212 μm at *λ* = 532 nm), a *xy*-localisation precision of a few nanometres is realised with *N* ∼ 10^4^ photons, corresponding to ∼10 s of the acquisition time for a typical fluorophore. Furthermore, for localisation in the *z* direction, several techniques to obtain nearly shot-noise-limited 3D localisation have been proposed by using a 2D imaging detector without scanning the sample or the laser focus^44^,^45^,^46^,^47^,^48^,^49^.

The unique cryogenic characteristics of the reflecting microscope (achromatism, high collection solid angle of 1.2 π sr, and high 3D spatial resolution) enable broad applications to not only cellular structures but also inorganic materials. Recently, using an aspherical reflecting objective, we succeeded in an optical detection of a single rare-earth ion in a bulk crystal^50^. Furthermore, the reflecting objective can be utilised at any temperature from ambient temperature to a few K, in any immersion conditions (air, water, and liquid nitrogen, etc), and for the wavelength ranging from 170 to 2,000 nm in which the material (fused silica) of the objective is transparent. Among the applications of our microscope under ambient conditions, one of the desires of biologists would be ultraviolet auto-fluorescence imaging of biological molecules that are localised on cellular structures.

## Methods

### Reflecting microscope

The *λ* = 532 nm (MDL-FN-532 nm-100 mW) and 635 nm (MLL-III-633 nm-50 mW) light sources were purchased from CNI laser and the *λ* = 780 nm (OBIS785LX-50 mW) light source was purchased from Coherent Inc. A plano-convex lens L1 (*f* = 100 mm) focused the three laser beams onto a light-source pinhole aperture (diameter of 20 μm). The diffracted light was perfectly collimated using an aluminium-coated concave mirror CM (*f* = 125 mm) and was reflected using a aluminium-coated mirror M1. CaF_2_ was chosen for a wedge window W1 that served as a beam splitter because of the low autofluorescence of the material. The collimated beams travelled to the inside of a superfluid-helium cryostat. The sample substrate in superfluid helium was moved using a piezo-driven cryo-scanner in the *xyz* directions (scanning range of 30 × 15 × 30 μm^3^ in the *x* × *y* × *z* directions, respectively; ANSxyz100, Attocube) and two cryo-positioners in the *xz* directions (travelling distance of 5 mm; ANPx101, Attocube). The *xyz* positions were monitored using three capacitive cryo-sensors that were custom-made for cryogenic experiments (detection range of 150 μm; Nanotex Corporation).

The sample positions were actively controlled using a feedback loop that consists of a piezo-driven cryo-scanner, capacitive cryo-sensors, and a homemade proportional-integral-derivative (PID) controller. Additional cryo-positioners were employed for coarse movements of 5 mm in the *xz* directions; the FOV in these directions was limited by the range of the cryo-sensors (150 μm). Furthermore, movement in the *y* direction was only performed using the piezo-driven cryo-scanner; the FOV in the *y* direction was limited by the scan range of the cryo-scanner (15 μm). Consequently, the FOV of the microscope was 150 × 15 × 150 μm^3^ in the *x* × *y* × *z* directions, respectively. If a *y* positioner with a travelling distance of a few millimetres is added to the sample holder, the FOV of the microscope in the *y* direction will be determined by the detection range of the cryo-sensor and will become (150 μm)^3^ in the *xyz* directions. The 780-nm light was detected using reflection from a CaF_2_ wedge window (W2), which was filtered using a band pass filter F1 (FBH780-10, Thorlabs), and was focused by an achromatic lens L2 (*f* = 300 mm, AC254-300-B, Thorlabs) onto a complementary metal-oxide semiconductor camera (ARTCAM-130SS-BW, ARTRAY).

With monitoring of the 780-nm laser spots on the camera, the aluminium-coated flat mirrors M2 and M3 could be used to align the incident direction of the excitation light with the direction of the principal ray of the reflecting objective because of the narrow FOV of the reflecting objective, as shown in Fig. 2c. In particular, the angle of M3 was finely controlled using piezo-driven actuators (NZA12, Newport). The excitation light at *λ*_ex_ = 532 and 635 nm was completely blocked by the F2 filters, a stack of two notch filters (NF01-633U-25, Semrock), and two long-pass filters (BLP01-532R-25, Semrock). The imaging lens L3 (*f* = 36 mm, MPLFLN5x, Olympus) coupled the fluorescence photons into a multimode fibre (mode field diameter of 50 μm, NA = 0.12; SFM-125Y, Fiberguide), and these photons were counted using an APD detector (Count^BLUE^, Laser Component).

### Reflecting objective

The dimensions of the aspherical reflecting objective with NA = 0.99 are shown in Fig. 1c. The reflecting objective consists of aspherical and spherical mirrors. These mirrors were formed by the vacuum deposition of aluminium onto polished fused-silica surfaces (SUPRASIL-P800, Shin-Etsu Quartz). The mirrors were protected with an over-coating of a thin SiO_2_ overcoat layer. The equation of the aspherical mirror (AM) is given in Fig. 1c, where *z* is the sag as a function of *r*, the reciprocal of the radius of the curvature *c* is (2.1 mm)^–1^, the conic constant *k* is –0.67, the second-order aspherical coefficient α_2_ is –5.981 × 10^–2^ mm^–1^, the fourth-order aspherical coefficient α_4_ is 1.022801 × 10^–4^ mm^–3^, the sixth-order aspherical coefficient α_6_ is –3.409849 × 10^–5^ mm^–5^, and the eighth-order aspherical coefficient α_8_ is –3.454710 × 10^–5^ mm^–7^. The surface irregularity of AM was *λ*/5 at a *λ* = 633 nm.

The spherical mirror (SM) with a radius *R*_SM_ = 15.000 mm was centred at *O*_SM_. The surface irregularity of SM was *λ*/14 at a *λ* = 633 nm. The decentration between AM and SM was less than 20 arcseconds. A spherical window (SW) with a radius of *R*_SW_ = 4.610 mm was centred at the focal point *O*. The dispersion of incident light due to the surface refraction of a plane window (PW) was cancelled by the surface refraction of SW. A single-layer MgF_2_ antireflection coating was applied to the surfaces of PW and SW. The reflectance of the antireflection coating in the range of 400 nm to 700 nm was less than 2%. The surface flatness of PW and SW were *λ*/23 and *λ*/8, respectively, at a *λ* = 633 nm. The specifications of the reflecting objective, the design of which was optimised using a Zemax simulation, are shown in Table 1. The focal length *f* was 2.09 mm, the maximum diameter of the parallel beam *ϕ*_a_ was 4.500 mm, the minimum diameter *ϕ*_b_ was 2.024 mm, the transmittance of the annular aperture of the entrance pupil *T*_eye_ was 0.8, the effective solid angle of the annular aperture lens *Ω* was 1.16 *π*sr, and the working distance (WD) was 1.0 mm. From the Zemax simulation, the energy within the first dark ring, *EE*_1st_, was estimated to be 0.33.

### Optimisation of the design of the reflecting objective

The structure was optimised in Zemax using the sequential mode. First, the curvatures of spherical mirror (SM) and aspherical mirror (AM) were optimised in vacuum. The initial condition of AM was set to a parabolic mirror having a focal length of 2 mm. Next, the complete design of the aspherical reflecting objective, which includes SW and PW, was optimised. The centre of the curvature of SW was set to the focal point *O*. The design made the chromatic aberration negligible because the refraction of SW is cancelled out by that of PW.

### Detection efficiency of the cryo-fluorescence microscope

Although the solid angle of the reflecting objective (Fig. 1a) was 1.16π, fluorescence photons of the dot were refracted at an interface between the polymer film and superfluid helium. Assuming the refractive index of the polymer film to be 1.5, the solid angle of collected fluorescence decreased to ∼0.3 π (0.08 of 4π). The internal transmittance of the reflecting objective was 0.74. The transmittance (T) of the three cryostat windows was 0.81. The T of the stain-less steel box consisting of two metallic mirrors, two CaF_2_ windows, and the CaF_2_ wedge plate (W1) was 0.76. The reflectance of the two dielectric mirrors was 0.99. The T of the CaF_2_ wedge plate (W2) was 0.94. The T of the four filters (F2) was 0.93. The T of the coupling lens (L3) was 0.92. The coupling efficiency of the multimode fibre was 0.92, and the quantum efficiency of the APD was 0.72. Overall, the total collection efficiency of the complete setup was 0.02.

### Sample preparation

A 10^–10^ M solution of a quantum dot (Qdot 705 carboxyl, Invitrogen) was prepared at pH = 7 in the presence of 20 mM phosphate buffer and 0.5% wt./wt. polyvinyl alcohol. The solution was spin-coated on a CaF_2_ substrate. To minimise the photo-blinking of the quantum dots, the sample was cooled to 1.7 K within 1 h after a stock solution of 8 mM was diluted.

## Additional Information

**How to cite this article**: Inagawa, H. *et al.* Reflecting microscope system with a 0.99 numerical aperture designed for three-dimensional fluorescence imaging of individual molecules at cryogenic temperatures. *Sci. Rep.* **5**, 12833; doi: 10.1038/srep12833 (2015).

## Figures and Tables

**Table 1:** Specifications of the optimised design of reflecting objectives in superfluid helium.

**NA**	***f*****/mm**	***ϕ***_**a**_**/mm**	***T***_**eye**_	***Ω*****/2***π***sr**	**WD/mm**	***EE***_**1st**_
0.99	2.09	4.500	0.80	0.58	1.0	0.33
0.58	2.00	2.250	0.75	0.13	6.0	0.42

The *EE*_1st_ values were obtained from the Zemax simulation results at *λ*_ex_ = 532 nm.

**Table 2:** Experimental observations and Zemax simulation results of the 1/e^2^ widths of the point spread functions (*Γ*) of aspherical and spherical reflecting objectives measured at 1.7 K.

**NA**	***λ***_**ex**_**/nm**	***Γ***_**xy**_**/**μ**m**		***Γ***_**z**_**/**μ**m**	
		**Observation (**σ)	**Simulation**	**Observation (**σ)	**Simulation**
0.99	532	0.212 ± 0.008	0.182	0.91 ± 0.04	0.65
0.99	635	0.238 ± 0.005	0.219	1.02 ± 0.02	0.78
0.58	532	0.394 ± 0.009	0.345	3.41 ± 0.07	3.13
0.58	635	0.436 ± 0.008	0.415	3.71 ± 0.06	3.66

The errors are one sigma deviations obtained from the Gaussian fitting analysis of five individual dots.
